# Flexible h-BN/GaN Heterostructure Thin-Film Piezoelectric Sensors for Harsh Environments

**DOI:** 10.3390/ma19173664

**Published:** 2026-08-28

**Authors:** Yi Peng, Wenwang Wei, Zhi Hu, Xiaolan Huang, Jianzhi Bai, Xifeng Xie, Qunsong He, Yang Zhou, Bei Huang, Zonghua Zhang, Lili Ding, Qiu Zhong, Lingyun Liu

**Affiliations:** 1 College of Electric Power Engineering, Guangxi Vocational College of Water Resources and Electric Power, 530023 Nanning, China; 2 Guangxi Key Laboratory of Calcium Carbonate Resources Comprehensive Utilization, College of Materials and Chemical Engineering, Hezhou University, Hezhou 542899, China

**Keywords:** h-BN/GaN heterostructure, piezoelectric sensor, piezotronic effect, free-carrier screening, harsh environments, high-temperature sensing, flexible inorganic electronics

## Abstract

Harsh-environment pressure sensing requires piezoelectric materials that can simultaneously withstand elevated temperature, mechanical loading, and structural degradation. GaN is a promising lead-free piezoelectric semiconductor owing to its wide bandgap, high thermal stability, and non-centrosymmetric wurtzite structure. However, its piezoelectric output can be significantly affected by free-carrier compensation in unintentionally n-type GaN. Here, we report a flexible all-inorganic piezoelectric pressure sensor based on a directly grown h-BN/GaN heterostructure thin film. The h-BN layer was deposited on GaN/Si by plasma-enhanced chemical vapor deposition, followed by backside Si removal, electrode deposition, and transfer onto a flexible Cu foil substrate. Structural characterizations confirmed the formation of a compact h-BN/GaN interface with clear lattice fringes, preferential out-of-plane orientation, and characteristic Raman signatures of both h-BN and GaN. Compared with the flexible GaN/Cu reference, the h-BN/GaN device exhibits modified interfacial electrical transport behavior, enhanced voltage and current-density outputs, and prolonged transient voltage retention. Finite-element simulations reveal modified electrostatic potential distribution after h-BN integration, while electrical and interfacial characterizations suggest electronic structure modulation and reduced carrier compensation effects at the heterointerface. Raman optothermal analysis indicates an improved relative/local thermal response of the h-BN/GaN device under identical optical excitation conditions, supporting its enhanced thermal robustness. Under 200 psi at 400 °C, the h-BN/GaN sensor maintains an output voltage of approximately 27.65 mV, about 2.32 times that of the GaN reference. This work demonstrates an interfacial engineering strategy based on two-dimensional h-BN integration for constructing flexible, thermally robust, and high-output piezoelectric sensors for harsh-environment monitoring.

## 1. Introduction

With the rapid development of aerospace, automotive, energy exploration, and deep-well drilling technologies, pressure sensors capable of stable operation under harsh thermal–mechanical environments are increasingly required [1,2,3,4]. Conventional piezoelectric sensors are still largely based on lead zirconate titanate (PZT) ceramics, but their long-term operation at elevated temperatures is restricted by depolarization, thermal instability near their Curie-temperature-related limits, and the environmental concerns associated with lead-containing materials [5,6,7]. Polymer-based piezoelectric materials, such as polyvinylidene fluoride (PVDF) and its copolymers, offer excellent flexibility and processability, but their relatively low melting point and limited thermal stability make them vulnerable to performance degradation and structural failure under high-temperature and high-load conditions [8,9,10]. Therefore, developing lead-free, thermally robust, and highly sensitive inorganic piezoelectric sensing materials remains an important challenge for next-generation harsh-environment sensors.

Gallium nitride (GaN), a representative third-generation wide-bandgap semiconductor, is an attractive candidate for high-temperature piezoelectric sensing because of its strong chemical inertness, high mechanical strength, large breakdown field, and excellent thermal stability [11,12,13]. Owing to its non-centrosymmetric wurtzite crystal structure, epitaxial GaN possesses spontaneous and piezoelectric polarization along the polar axis, enabling electromechanical conversion without the high-voltage poling process required for many ferroelectric ceramics and polymers [14,15]. However, the sensing performance of GaN-based piezoelectric devices is often limited by an intrinsic free-carrier screening effect [16,17]. As-grown GaN typically exhibits n-type conductivity because of residual donors and native defects, such as nitrogen vacancies [18,19]. Under mechanical stress, the strain-induced piezoelectric polarization charges can be rapidly compensated by free electrons, thereby reducing the effective piezopotential and weakening the macroscopic electrical output of the device [20].

Constructing semiconductor heterojunctions provides an effective strategy to suppress carrier screening and enhance the piezoelectric response through band engineering [20,21]. Complementary optical approaches, particularly absorption-coefficient analysis, can also provide useful information on band-edge transitions, optical gaps, and defect-related states, further broadening the characterization tools available for band-engineered semiconductor systems [22,23]. In p-n or barrier-type heterojunctions, the built-in electric field and depletion region can reduce the concentration of mobile carriers near the interface, thereby preserving strain-induced piezoelectric charges [24]. More importantly, according to the piezotronic effect, these piezoelectric polarization charges can act as an internal gate voltage to dynamically modulate the interfacial band bending and barrier height [25]. As a result, the carrier transport across the heterointerface can be strongly regulated by mechanical deformation, leading to a much larger electrical response than that obtained from conventional piezoresistive modulation alone.

Hexagonal boron nitride (h-BN) is a wide-bandgap two-dimensional material with excellent thermal stability, chemical resistance, electrical insulation, and mechanical robustness, making it highly suitable for constructing all-inorganic heterostructures for harsh-environment sensing [26,27]. Beyond its chemical and thermal robustness, h-BN is also advantageous for flexible piezoelectric devices because it can act as a mechanically stable interfacial layer to help release residual stress and suppress local strain concentration after substrate removal. Its high thermal stability and in-plane heat-spreading capability are also beneficial for reducing heat accumulation in the active GaN layer under high-temperature operation [28]. More importantly, integrating h-BN with n-type GaN offers a potential interfacial band-engineering strategy [29], in which a barrier-type heterointerface may regulate carrier redistribution and reduce the rapid compensation of strain-induced polarization charges. Such an interfacial design is expected to help preserve the piezopotential generated in GaN and thereby improve the piezoelectric output. However, the integration of h-BN with GaN remains challenging because conventional high-temperature epitaxial growth or post-transfer assembly may introduce lattice/thermal-mismatch-induced defects, interfacial contamination, wrinkles, trapped air gaps, and nonuniform stress transfer [30,31,32]. To address these issues, plasma-enhanced chemical vapor deposition (PECVD) provides a feasible route for directly growing h-BN thin films on GaN at relatively low temperatures. The plasma-assisted growth process promotes the formation of conformal h-BN layers on the GaN surface, improving interfacial contact and enabling efficient electromechanical coupling across the h-BN/GaN interface without the need for polymer-assisted stacking. Furthermore, after direct h-BN growth, the heterostructure thin film can be released from the rigid substrate through a controlled lift-off process to form a flexible, all-inorganic sensing membrane, thereby combining strong interfacial coupling with mechanical flexibility and high-temperature robustness.

Herein, we propose and experimentally demonstrate a high-performance piezoelectric pressure sensor based on an all-inorganic h-BN/GaN heterostructure thin film directly integrated by PECVD growth and substrate release. The directly grown h-BN/GaN interface is designed to introduce a barrier-type interfacial region that may mitigate free-carrier compensation in n-type GaN and help preserve the strain-induced piezopotential through piezotronic band modulation. Compared with polymer-assisted or single-component piezoelectric sensors, the h-BN/GaN device combines the intrinsic piezoelectricity and high-temperature stability of GaN with the thermal robustness, insulating nature, and heat-spreading capability of h-BN. Benefiting from this synergistic interfacial engineering and all-inorganic flexible architecture, the sensor exhibits enhanced output voltage, good pressure-dependent linearity, improved thermal dissipation, and stable operation under elevated temperatures up to 400 °C and applied pressures up to 200 psi. This work provides a promising route for developing directly integrated all-inorganic piezotronic sensors for harsh-environment monitoring.

## 2. Materials and Methods

Commercial 2-inch GaN-on-Si wafers were used as the starting substrates for the fabrication of the h-BN/GaN heterostructure sensors. The wafers consisted of a 430 μm thick undoped single-side-polished Si substrate and a 1 μm unintentionally doped GaN epitaxial layer. Although no intentional dopants were introduced during GaN growth, Hall-effect measurements confirmed that the GaN layer exhibited weak n-type conductivity, with an electron concentration of approximately 5 × 10^16^ cm^−3^. This residual n-type behavior is mainly attributed to donor-like native defects and unintentional impurities, such as nitrogen vacancies, oxygen-related impurities, and possible Si incorporation during epitaxial growth [33].

**Figure 1:**
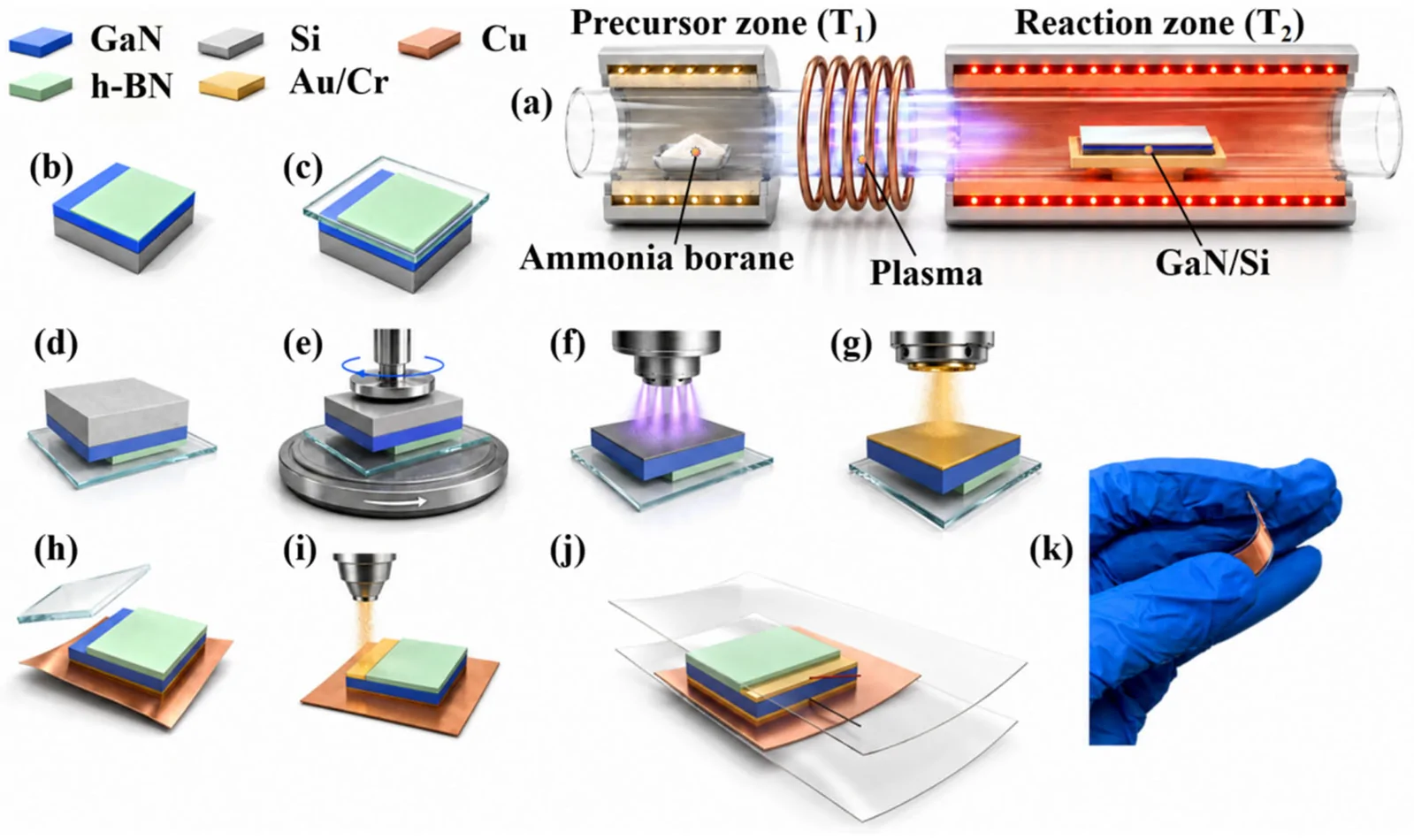
Schematic illustration of the fabrication process of the flexible h-BN/GaN/Cu piezoelectric device. (**a**) PECVD growth of h-BN on the GaN/Si substrate. (**b**) Formation of the h-BN/GaN/Si heterostructure with a reserved region for top-electrode deposition. (**c**) Temporary bonding to a transparent carrier. (**d**) Flipping of the bonded sample. (**e**) Backside mechanical thinning of the Si substrate. (**f**) Si removal by DRIE followed by XeF_2_ dry release. (**g**) Deposition of the Cr/Au bottom electrode on the exposed GaN backside. (**h**) Removal of the temporary carrier and transfer onto a flexible Cu foil. (**i**) Deposition of the Au/Cr top electrode. (**j**) Wire connection and encapsulation with a flexible transparent protective layer. (**k**) Photograph of the final flexible h-BN/GaN/Cu device. A reference flexible GaN/Cu device used for comparison was fabricated through the same substrate-removal, electrode-fabrication, and Cu-foil-transfer procedure, with the h-BN integration step omitted.

The fabrication process of the flexible h-BN/GaN/Cu device is shown in Figure 1a–k. First, an h-BN film was directly grown on a GaN/Si substrate by PECVD using ammonia borane (NH_3_BH_3_) as the precursor (Figure 1a,b). The precursor was heated to 90–110 °C in the upstream zone and transported by N_2_ at 40 sccm to the downstream reaction zone with a temperature of 800 °C, where plasma-assisted decomposition, adsorption, and dehydrogenation led to the growth of h-BN on GaN.

The obtained h-BN/GaN/Si sample was then temporarily bonded to a rigid transparent carrier (Figure 1c) and flipped over (Figure 1d). The backside Si was first mechanically thinned (Figure 1e), followed by DRIE to remove most of the Si and XeF_2_ dry etching to release the residual Si (Figure 1f). After substrate removal, a Cr/Au bottom electrode was deposited on the exposed backside of GaN by magnetron sputtering (Figure 1g). The temporary carrier was subsequently removed, and the released thin film was transferred onto a flexible Cu foil substrate (Figure 1h). A top Cr/Au electrode was then sputtered onto the reserved region on the upper surface of the heterostructure (Figure 1i). Finally, lead wires were connected to the top and bottom electrodes, and the device was encapsulated with a flexible transparent protective layer (Figure 1j), yielding the final flexible h-BN/GaN/Cu device (Figure 1k).

For comparison, a reference flexible GaN/Cu device without the h-BN layer was fabricated using the same device-processing procedure. The GaN reference underwent the same temporary bonding, backside Si mechanical thinning, DRIE and XeF_2_-assisted Si removal, Cr/Au bottom-electrode deposition, release from the temporary carrier, transfer onto flexible Cu foil, top-electrode deposition, and wire-connection steps as the h-BN/GaN device. Thus, both devices experienced the same substrate-release, flexible-transfer, electrode-fabrication, and associated stress-relaxation processes, with the presence or absence of the h-BN layer being the principal structural difference between the two device configurations. The GaN reference and h-BN/GaN devices were subsequently evaluated using the same electrical and pressure-testing procedures.

Finite-element simulations were performed using COMSOL Multiphysics 6.3 to evaluate the electrostatic/piezopotential distributions of the GaN and h-BN/GaN structures under pressure loading and elevated-temperature conditions. A representative three-dimensional model with a lateral dimension of 100 μm × 100 μm was constructed. The GaN layer was 1.0 μm thick, and a 0.2 μm-thick h-BN layer was introduced in the heterostructure model. GaN was treated as the dominant piezoelectric layer, whereas h-BN was modeled as an anisotropic dielectric interfacial layer. The relative permittivity components were set as (ε_x_, ε_y_, ε_z_) = (9.5, 9.5, 10.4) for GaN and (6.93, 6.93, 3.76) for h-BN. For GaN, the c-axis elastic constant and piezoelectric stress constant were set as C_33_ = 398 GPa and e_33_ = 0.73 C∙m^−2^, respectively. More details can be found in Table 1.

The experimental mechanical loading was represented by a spatially uniform nominal pressure applied normal to the modeled device surface. The same pressure condition was used for the GaN and h-BN/GaN structures to enable a controlled comparison. Under the uniform c-axis approximation, the pressure-induced GaN strain and polarization charge density were evaluated using ε_zz_ = P/C_33_ and σ_pz_ = e_33_ε_zz_, respectively. In the electrostatic model, the bottom electrode was grounded, the external bias was set to zero, and the pressure-induced potential was applied through the GaN piezoelectric response. The output voltage was obtained from the potential difference between the top and bottom electrodes, while the remaining external boundaries were treated as electrically insulating. This mechanical treatment represents an average-pressure approximation and does not explicitly resolve local contact-pressure variations, lateral stress redistribution, or edge-induced stress concentrations.

Temperature-dependent simulations were performed over 298.15–673.15 K using first-order corrections to the dielectric and electromechanical parameters. The temperature-correction coefficients adopted in the model were 1.0 × 10^−4^ K^−1^ and 3.0 × 10^−4^ K^−1^ for the dielectric responses of h-BN and GaN, respectively, and 7.0 × 10^−5^ K^−1^ and 1.5 × 10^−4^ K^−1^ for C_33_ and e_33_, respectively. These coefficients were used as first-order modeling parameters to describe the temperature evolution of the dielectric and electromechanical response. A free tetrahedral mesh and stationary solver were used. Mesh refinement was performed to verify numerical convergence of the extracted potential. The present model focuses on the electrostatic potential redistribution and electromechanical response induced by h-BN integration. The interfacial carrier modulation mechanism is further evaluated through the combined electrical characterization and XPS analysis.

Pressure-dependent piezoelectric measurements were performed using a calibrated mechanical loading system. The applied force was calibrated using a force gauge, and the nominal pressure was calculated by dividing the measured normal force by the effective device area (1 cm^2^). At room temperature, cyclic compression was applied using a DC-motor-driven reciprocating actuator equipped with a rigid 8 cm × 10 cm rectangular platen, which completely covered the 1 cm × 1 cm device surface. Prior to the piezoelectric measurements, the motor-driven loading system was calibrated using a force gauge positioned at the device location, such that the normal force corresponding to each actuator setting was experimentally determined rather than inferred from the actuator displacement. The nominal pressure was calculated as $P_{nom}=F/A_{dev}$, where (F) is the calibrated normal force and $A_{dev}=1.0\;cm^{2}$ is the projected device area. The actuator was operated over a 60 mm stroke with maximum extension and retraction velocities of $0.2\;m\cdot s^{-1}$ and acceleration/deceleration settings of $2\;m\cdot s^{-2}$, corresponding to an estimated compression–release cycle frequency of approximately 1.25 Hz under the programmed motion conditions. A total of 1000 compression–release cycles were performed during the room-temperature cyclic-loading test. For high-temperature measurements, the motor-driven loading apparatus was replaced by direct force-gauge loading to accommodate the heating stage. A flat circular loading head with a diameter of 1.5 cm was used. Because the loading head was also larger than the 1 cm × 1 cm device and completely covered its surface, the same effective device area of $1.0\;cm^{2}$ was used to calculate the nominal pressure from the directly measured normal force. A total of 100 compression–release cycles were recorded during the high-temperature measurements without a fixed actuator-controlled loading frequency. The reported pressures therefore represent nominal average pressures over the projected device area, while local deviations from uniform pressure may occur because of contact alignment, device compliance, surface flatness, edge effects, and the flexible Cu support.

## 3. Results and Discussion

The electrical transport and piezoelectric output characteristics of the h-BN/GaN device were systematically compared with those of the matched released/flexible GaN/Cu reference device fabricated using the same substrate-removal, transfer, and electrode-fabrication processes but without the h-BN layer. Consequently, effects associated with Si-substrate removal, flexible transfer, and process-induced residual-stress relaxation were common to both devices, enabling a more direct evaluation of the effect of h-BN integration.

**Figure 2:**
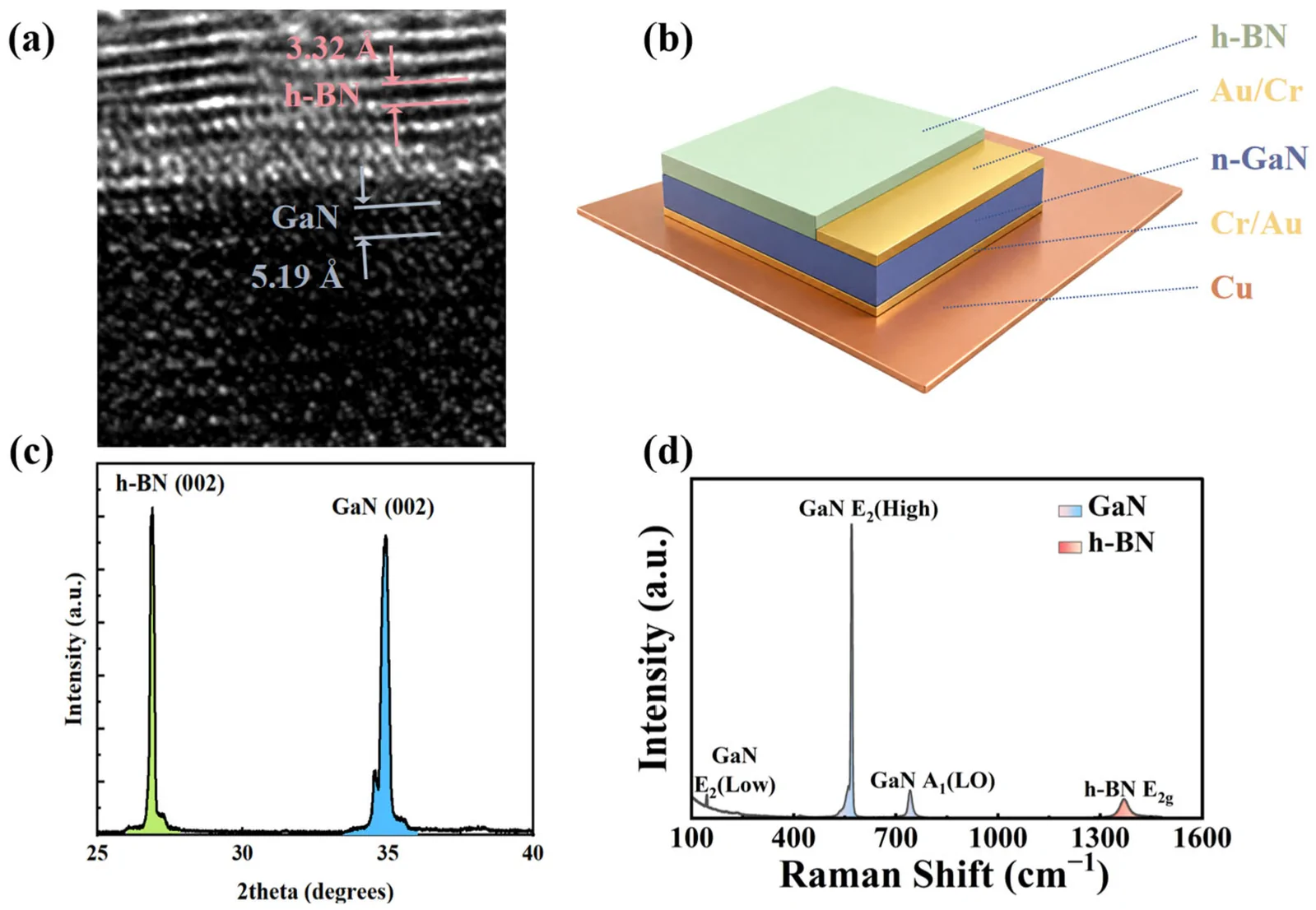
Structural characterization of the h-BN/GaN heterostructure. (**a**) Cross-sectional HRTEM image of the h-BN/GaN interface, showing a continuous layered h-BN film on GaN with measured lattice spacings of 3.32 Å for h-BN and 5.19 Å along the GaN c-axis direction. (**b**) Schematic illustration of the h-BN/GaN/Cu device structure, consisting of h-BN, Au/Cr top electrode, n-GaN, Cr/Au bottom electrode, and flexible Cu substrate. (**c**) XRD 2θ–ω scan showing the h-BN (002) and GaN (002) diffraction peaks, indicating the coexistence of crystalline h-BN and c-axis-oriented GaN with a preferential out-of-plane orientational relationship. (**d**) Raman spectrum of the h-BN/GaN heterostructure, showing the characteristic vibrational modes of GaN and h-BN.

The formation of the h-BN/GaN heterostructure was first examined by cross-sectional high-resolution transmission electron microscopy (HRTEM). As shown in Figure 2a, a layered h-BN film is clearly observed on the GaN surface, forming a sharp and continuous interface with the underlying GaN. No obvious interfacial voids, cracks, or delamination can be observed, suggesting that the PECVD-grown h-BN is conformally integrated with the GaN layer. Such intimate interfacial contact is particularly important for piezoelectric devices, because efficient stress transfer and interfacial charge coupling strongly depend on the mechanical and electrical continuity across the heterointerface [34].

The lattice-resolved HRTEM image further reveals ordered lattice fringes in both the h-BN and GaN regions. The interlayer spacing of the upper layered region is measured to be approximately 3.32 Å, which agrees well with the characteristic spacing of the h-BN (002) planes [35]. This confirms the formation of layered h-BN with its basal planes preferentially aligned parallel to the GaN surface. In the GaN region, a lattice periodicity of approximately 5.19 Å is observed along the c-axis direction, consistent with the wurtzite GaN lattice [36]. These results indicate that the h-BN layer was successfully grown on GaN while maintaining a well-defined crystalline interface.

Figure 2b illustrates the device architecture constructed from the h-BN/GaN heterostructure. The device consists of a top h-BN layer, a reserved Au/Cr top electrode on the exposed GaN region, an n-type GaN piezoelectric layer, a Cr/Au bottom electrode, and a flexible Cu supporting substrate. In this configuration, GaN serves as the main piezoelectric semiconductor layer, while the h-BN layer provides a thermally robust and electrically insulating interfacial layer. The combination of h-BN and GaN is expected to suppress free-carrier screening at the GaN surface and enhance the stability of strain-induced polarization charges, which provides the structural basis for the subsequent piezoelectric sensing performance.

The crystallographic orientation of the h-BN/GaN heterostructure was further investigated by X-ray diffraction in the 2θ–ω mode, as shown in Figure 2c. Two dominant diffraction peaks assigned to h-BN (002) and GaN (002) are observed, confirming the coexistence of crystalline h-BN and GaN. The presence of the h-BN (002) reflection indicates that the h-BN film possesses a preferential c-axis orientation, with its basal planes parallel to the GaN surface. Meanwhile, the strong GaN (002) peak confirms the c-axis-oriented wurtzite GaN layer. The simultaneous observation of h-BN (002) and GaN (002) reflections suggests a well-defined out-of-plane orientational relationship between the two materials, consistent with the lattice-resolved HRTEM results.

Raman spectroscopy was employed as a complementary technique to verify the chemical and vibrational signatures of the heterostructure. As shown in Figure 2d, the spectrum exhibits the characteristic Raman modes of GaN, including the E_2_(low), E_2_(high), and A_1_(LO) modes, indicating that the GaN lattice remains well preserved after PECVD growth of h-BN. In addition, the h-BN E_2g_ mode is observed at high wavenumber, providing further evidence for the successful formation of h-BN on the GaN surface. The coexistence of GaN and h-BN Raman features, together with the HRTEM and XRD results, confirms the successful construction of a structurally integrated h-BN/GaN heterostructure.

Overall, the combined HRTEM, XRD, and Raman characterizations demonstrate that crystalline h-BN was successfully grown on GaN by PECVD. The resulting heterostructure exhibits a compact interface, clear lattice features, and preferential out-of-plane orientation, which are essential prerequisites for efficient electromechanical coupling and stable operation of the flexible h-BN/GaN piezoelectric device.

**Figure 3:**
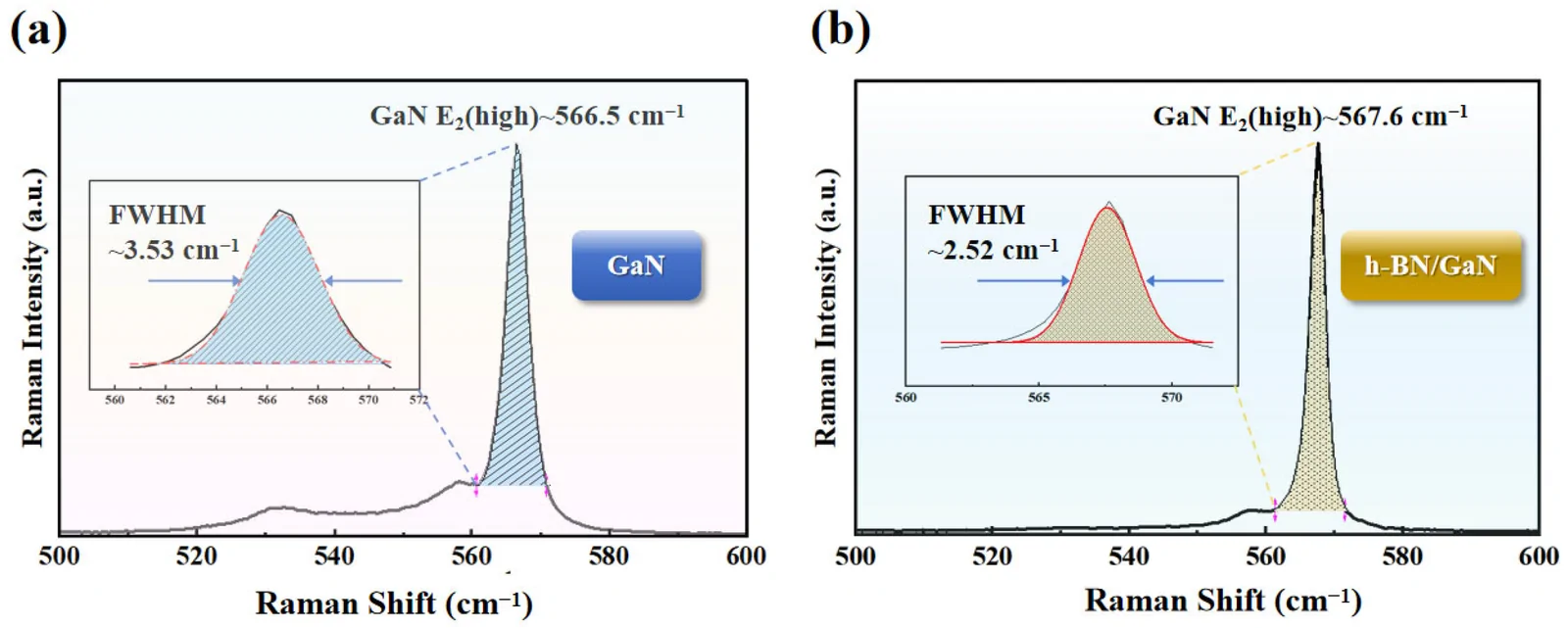
Raman analysis of the GaN E_2_(high) mode before and after device fabrication. (**a**) Raman spectrum of the original GaN/Si substrate, showing the GaN E_2_(high) peak at approximately 566.5 cm^−1^ with a FWHM of ~3.53 cm^−1^. The red shift relative to stress-free GaN indicates residual tensile stress in the as-grown GaN layer. (**b**) Raman spectrum of the fabricated h-BN/GaN/Cu device, showing the GaN E_2_(high) peak shifted to approximately 567.6 cm^−1^ with a reduced FWHM of ~2.52 cm^−1^, suggesting effective stress relaxation and reduced strain inhomogeneity after Si removal and flexible device integration.

Raman spectroscopy was further employed to evaluate the strain state of GaN before and after device fabrication, as shown in Figure 3. The nonpolar E_2_(high) phonon mode of wurtzite GaN is highly sensitive to residual stress and is therefore commonly used as a fingerprint for strain evaluation. For stress-free GaN, the E_2_(high) mode is generally located at approximately 567.5 cm^−1^ [37]. In the as-grown GaN/Si substrate, the E_2_(high) peak appears at approximately 566.5 cm^−1^, as shown in Figure 3a. This red shift relative to the stress-free position indicates that the GaN layer grown on Si is subjected to residual tensile stress, which can be attributed to lattice mismatch, thermal expansion mismatch, and the mechanical constraint imposed by the rigid Si substrate.

After the fabrication of the flexible h-BN/GaN/Cu device, the GaN E_2_(high) mode shifts to approximately 567.6 cm^−1^ as shown in Figure 3b. The peak position becomes very close to that of stress-free GaN, suggesting that the residual stress in the GaN layer is significantly released during the backside Si removal and flexible transfer process. This stress relaxation is mainly associated with the elimination of the rigid Si substrate constraint. Meanwhile, the conformal h-BN layer and the flexible Cu support may help maintain the structural integrity of the released GaN thin film and suppress local stress concentration during transfer and bending.

In addition to the peak-position shift, the full width at half maximum (FWHM) of the GaN E_2_(high) mode decreases from approximately 3.53 cm^−1^ for the original GaN substrate to approximately 2.52 cm^−1^ for the h-BN/GaN device. The narrower Raman linewidth indicates reduced phonon scattering and suppressed inhomogeneous strain broadening in the released heterostructure. This result suggests that the fabrication process not only preserves the crystalline lattice of GaN but also improves the local strain uniformity of the active piezoelectric layer. Such stress relaxation and structural homogenization are beneficial for piezoelectric sensing, because excessive residual stress and strain fluctuation can disturb the piezoelectric potential distribution and degrade the stability of the output signal [38].

Therefore, the Raman results demonstrate that the conversion from the rigid GaN/Si substrate to the flexible h-BN/GaN/Cu device effectively releases the residual tensile stress in GaN while maintaining a high-quality lattice response. The h-BN/GaN heterostructure thus provides a mechanically stable platform for subsequent flexible and high-temperature piezoelectric sensing.

**Figure 4:**
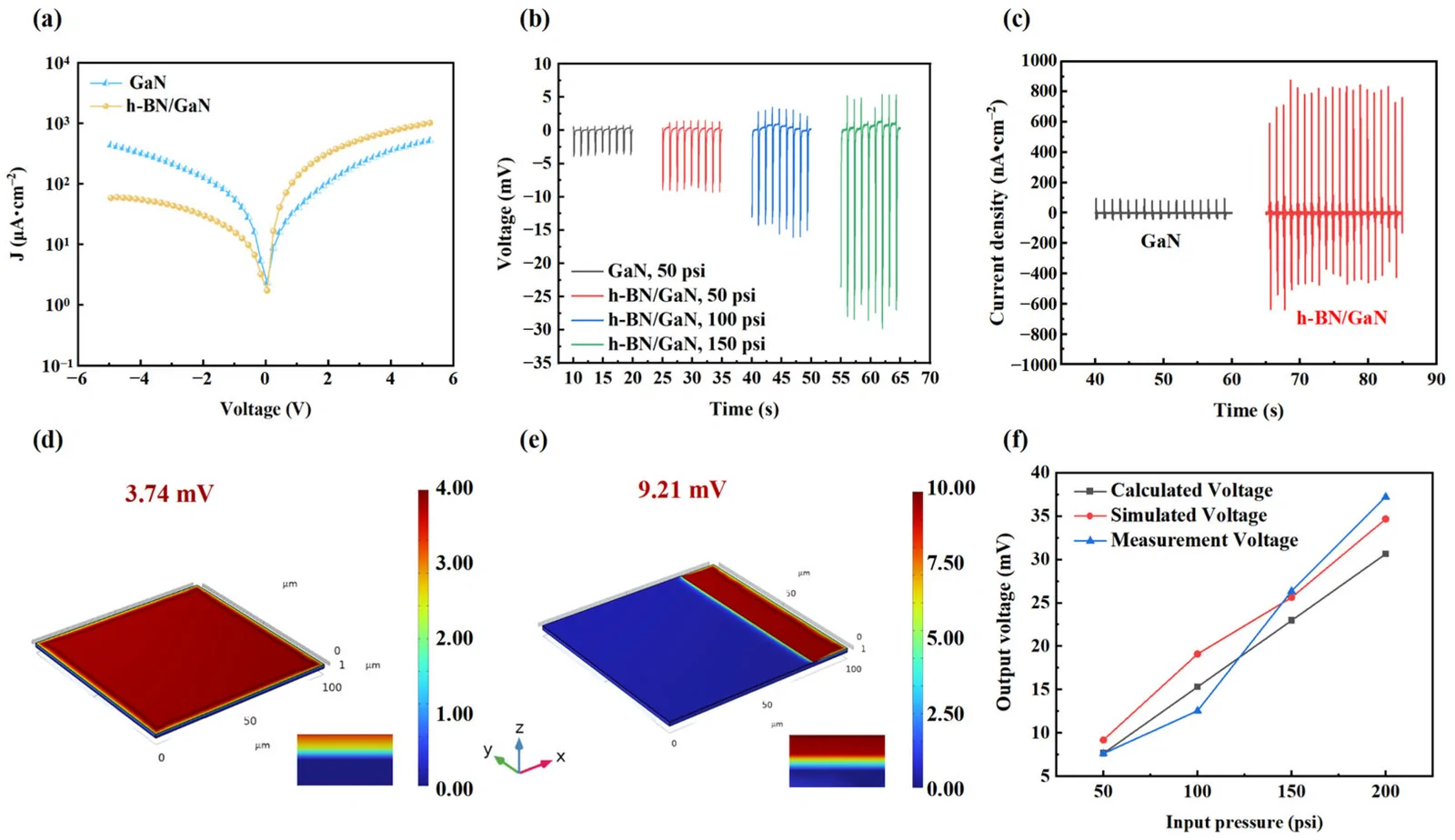
Electrical and piezoelectric performance of the h-BN/GaN device. (**a**) J–V characteristics of the matched flexible GaN/Cu reference and h-BN/GaN devices. (**b**) Dynamic output-voltage responses under periodic pressure loading. (**c**) Comparison of transient output current-density responses. (**d**,**e**) COMSOL-calculated electrostatic/piezopotential distributions of the GaN and h-BN/GaN structures under identical nominal mechanical loading; the model describes dielectric/electrostatic field redistribution and does not explicitly include mobile-carrier screening. (**f**) Comparison of analytical, simulated, and experimentally measured output voltages of the h-BN/GaN device as a function of input pressure.

The electrical transport and piezoelectric output characteristics of the h-BN/GaN device were systematically evaluated and compared with those of a reference GaN device without the h-BN layer. Figure 4a shows the current-density–voltage (J–V) characteristics of the two devices. Compared with the flexible GaN/Cu reference, the h-BN/GaN device exhibits a more asymmetric J–V behavior under positive and negative bias conditions. This non-ohmic transport behavior indicates modified interfacial electrical characteristics after the introduction of h-BN. The wide-bandgap and electrically insulating nature of h-BN can reduce direct carrier transport across the GaN surface and contribute to suppressed leakage under reverse or low-bias conditions. Meanwhile, the enhanced current density under forward bias suggests that the interfacial electronic structure is influenced by the applied electric field, resulting in transport behavior different from simple ohmic conduction.

Such asymmetric transport characteristics suggest the presence of an electrically modulated h-BN/GaN interface, which may influence carrier redistribution and charge compensation processes during piezoelectric operation. However, the J–V asymmetry alone does not uniquely determine the microscopic transport mechanism, as contributions from interface states, contact effects, and other non-ohmic transport processes cannot be fully excluded. Therefore, the role of the h-BN interface in reducing carrier compensation and preserving strain-induced piezoelectric polarization charges is further discussed based on the combined evidence from XPS-derived electronic structure analysis, electrical characterization, and piezoelectric output measurements.

The piezoelectric voltage response was then measured under periodic pressure loading, as shown in Figure 4b. Under the same pressure of 50 psi, the h-BN/GaN device produces a significantly larger output voltage than the reference GaN device. This enhancement indicates that the incorporation of h-BN effectively improves the electromechanical conversion capability of the GaN-based sensor. For the h-BN/GaN device, the output voltage increases progressively as the applied pressure rises from 50 to 150 psi, demonstrating a clear pressure-dependent response. The stable and repeatable voltage pulses under each pressure condition also indicate good mechanical reliability and reversible piezoelectric response of the flexible heterostructure device.

The output current-density response further confirms the enhanced piezoelectric performance after h-BN integration. As shown in Figure 4c, the h-BN/GaN device exhibits a much larger transient current-density signal than the GaN reference under dynamic loading. Since piezoelectric current originates from the redistribution of strain-induced polarization charges during loading and unloading, the higher current output suggests that more effective piezoelectric charges are preserved and collected in the h-BN/GaN device. This improvement can be attributed to the combined effects of the interfacial barrier, reduced free-carrier screening in GaN, and enhanced charge separation at the h-BN/GaN interface.

To further understand the influence of h-BN integration on the electromechanical response, finite-element simulations were performed for the GaN and h-BN/GaN structures under identical pressure loading conditions. As shown in Figure 4d,e, the simulated piezopotential of the h-BN/GaN heterostructure is higher than that of the GaN reference, increasing from approximately 3.74 mV to 9.21 mV.

Within the framework of the present model, this enhancement originates from the modified dielectric boundary condition and electrostatic potential redistribution introduced by the anisotropic h-BN interfacial layer. Compared with GaN (ε_z_ = 10.4), h-BN exhibits a lower out-of-plane dielectric constant (ε_z_ = 3.76), which modifies the vertical electric-field distribution and potential allocation within the heterostructure. Therefore, the h-BN layer contributes to improved electrostatic potential retention under mechanical excitation.

Although the present electrostatic model does not explicitly describe dynamic carrier transport, interface-state evolution, or time-dependent charge redistribution, the simulated potential variation provides complementary insight into the role of the h-BN interface. Combined with the XPS, electrical, and transient measurements, these results support the proposed interfacial modulation mechanism responsible for the enhanced piezoelectric response.

Figure 4f compares the analytical calculation, finite-element simulation, and experimental output voltages under different pressure conditions. All three results exhibit an approximately linear increase with increasing pressure, indicating that the analytical model and finite-element simulation capture the first-order pressure-dependent electromechanical behavior of the device.

The slight deviations between the calculated, simulated, and experimental values originate from the idealized uniform-pressure assumption, practical stress-transfer conditions, electrode effects, and interfacial nonidealities. Nevertheless, the consistent pressure-dependent trend demonstrates that the h-BN/GaN heterostructure maintains an enhanced piezoelectric response compared with the GaN reference.

According to the simplified parallel-plate capacitor model reported in previous studies, the output voltage of a piezoelectric film under uniform pressure can be expressed as [13]:

(1) $$V_{out}=\frac{\sigma A}{C}=\frac{e\frac{P}{E}A}{\frac{k\varepsilon_{0}A}{t}}=\frac{ePt}{k\varepsilon_{0}E}$$

where *V_out_* is the output voltage, σ is the pressure-induced surface charge density, A is the effective electrode area, *C* is the capacitance of the piezoelectric film, e is the piezoelectric coefficient, *P* is the applied pressure, *E* is the Young’s modulus, *k* is the relative dielectric constant, *ε*_0_ is the vacuum permittivity, and *t* is the film thickness. In this model, the pressure-induced strain is approximated as *P*/*E*, and the capacitance is given by *C = kε*_0_*A*/*t*.

For the present h-BN/GaN heterostructure device, GaN is regarded as the dominant piezoelectric layer. Since the top electrode is directly deposited on the GaN surface, the h-BN layer is not treated as an additional dielectric layer connected in series with GaN. To retain the compact scalar form of Equation (1) while accounting for the anisotropic out-of-plane electromechanical response of c-axis-oriented wurtzite GaN, an effective anisotropy correction factor, η_eff_, is introduced:

(2) $$V_{out}=\eta_{eff}\frac{e_{eff}Pt_{GaN}}{\varepsilon_{0}\varepsilon_{r,GaN}E_{GaN}}$$

where *e_eff_*, *t_GaN_*, *ε_r,GaN_*, and *E_GaN_* represent the effective piezoelectric coefficient, GaN thickness, scalar relative dielectric constant, and scalar Young’s modulus used in the simplified analytical model, respectively. The correction factor *η_eff_* was independently estimated from the anisotropic elastic and dielectric properties of wurtzite GaN according to

(3) $$\eta_{eff}=\frac{E_{GaN}}{E_{c}}\frac{\varepsilon_{r,GaN}}{\varepsilon_{33}}$$

where *E_c_* and *ε*_33_ are the effective Young’s modulus and relative permittivity along the c-axis, respectively. For hexagonal wurtzite GaN, the effective c-axis Young’s modulus can be expressed as

(4) $$E_{c}=C_{33}-\frac{2C_{13}^{2}}{C_{11}+C_{12}}$$

Using the experimentally reported elastic constants of wurtzite GaN, *C*_11_ = 390 GPa, *C*_12_ = 145 GPa, *C*_13_ = 106 GPa, and *C*_33_ = 398 GPa [39], the effective c-axis Young’s modulus is calculated to be *E_c_* ≈ 356 GPa. The out-of-plane relative permittivity of GaN was taken as *ε*_33_ = 10.4, consistent with reported anisotropic dielectric data for wurtzite GaN [40]. Relative to the scalar reference values *E_GaN_* = 2.5 × 10^11^ Pa, and ε_r,GaN_ = 8.9 used in the simplified analytical model, the resulting anisotropy correction factor is

(5) $$\eta_{eff}=\frac{E_{GaN}}{E_{c}}\frac{\varepsilon_{r,GaN}}{\varepsilon_{33}}=\frac{250}{356}\times \frac{8.9}{10.4}\approx 0.60$$

Therefore, *η_eff_* = 0.60 was adopted in the analytical calculation. This value was determined from the independently reported anisotropic elastic and dielectric parameters of GaN.

In the calculation, the following parameters were used: *e_eff_* = 0.73 C m^−2^, *t_GaN_* = 1.0 μm, *ε*_0_ = 8.854 × 10^−12^ F·m^−1^, *ε_r,__GaN_* = 8.9, and *E_GaN_* = 2.5 × 10^11^ Pa [41]. Considering the non-ideal characteristics of the practical device, η_eff_ was taken as 0.60. Under applied pressures of 50, 100, 150, and 200 psi, the calculated output voltages were 7.66, 15.33, 22.99, and 30.66 mV, respectively. The analytical model is used as a first-order description of the pressure-dependent piezoelectric output and is not intended to explicitly describe free-carrier screening or interfacial carrier dynamics.

Taken together, the electrical characterization, dynamic piezoelectric measurements, and simulation results demonstrate that the introduction of h-BN significantly enhances the output performance of GaN-based piezoelectric sensors. The h-BN layer not only forms a barrier-type interface that suppresses free-carrier screening in n-GaN, but also improves interfacial charge separation and piezopotential preservation. These effects collectively contribute to the higher voltage output, larger current-density response, and good pressure-dependent linearity of the flexible h-BN/GaN/Cu piezoelectric device.

**Figure 5:**
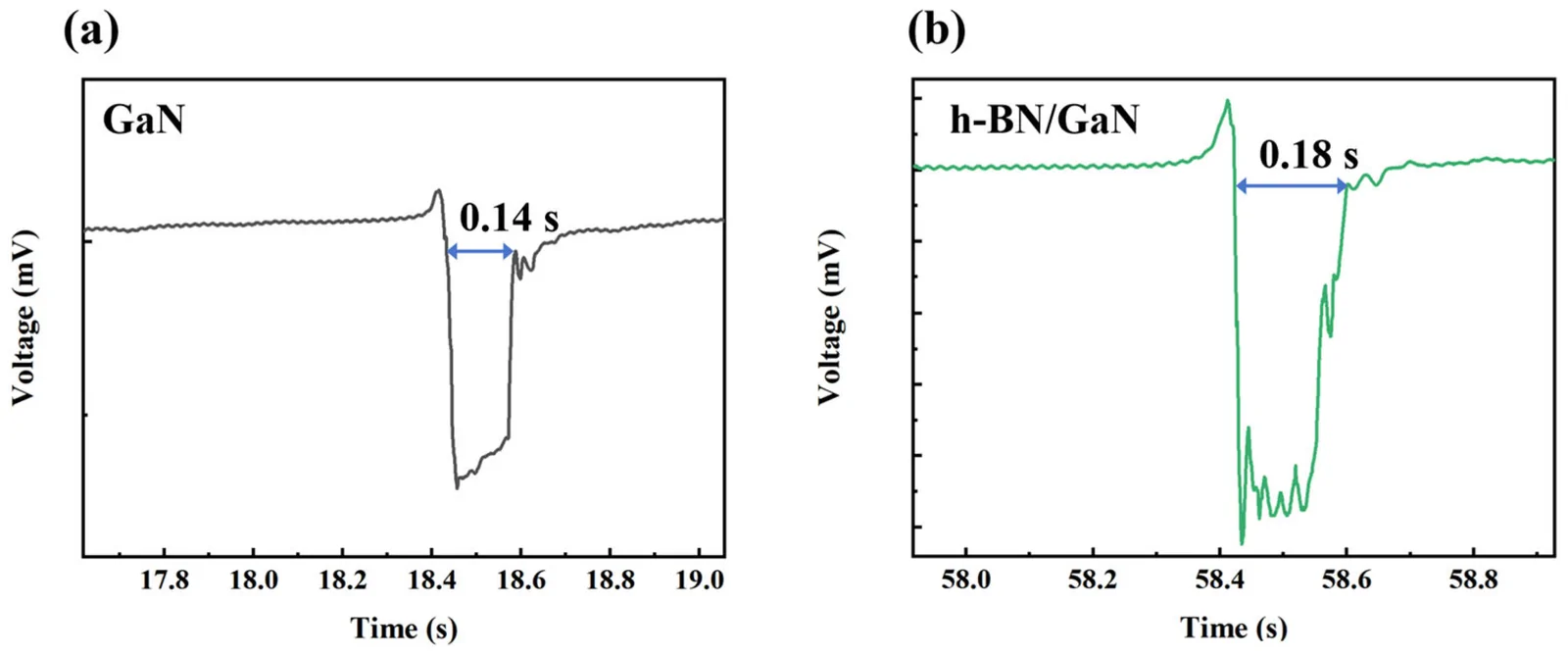
Enlarged transient voltage responses of the GaN and h-BN/GaN devices. (**a**) Local magnification of an output-voltage pulse from the reference GaN device, showing a characteristic voltage duration of approximately 0.14 s. (**b**) Local magnification of an output-voltage pulse from the h-BN/GaN device, showing an extended voltage duration of approximately 0.18 s. The prolonged transient voltage duration of the h-BN/GaN device indicates enhanced piezopotential retention and modified interfacial charge dynamics after h-BN integration.

To further compare the transient voltage characteristics of the GaN and h-BN/GaN devices, enlarged views of individual output-voltage pulses were analyzed, as shown in Figure 5. For the reference GaN device, the voltage signal rapidly decays after mechanical stimulation, with a characteristic pulse duration of approximately 0.14 s (Figure 5a). The relatively short voltage-retention time indicates a rapid decay of the strain-induced piezoelectric potential after the removal of external loading, which is consistent with efficient charge compensation processes in n-type GaN.

In contrast, the h-BN/GaN device exhibits a longer transient voltage duration of approximately 0.18 s under the same testing conditions (Figure 5b). The extended voltage-retention behavior suggests that the piezoelectric potential generated in GaN can be maintained for a longer period after h-BN integration. This enhanced charge-retention behavior is attributed to the modified interfacial electrical environment introduced by the h-BN layer, which may influence carrier redistribution and charge compensation processes at the heterointerface. In addition, the insulating h-BN layer may modify the effective capacitance and equivalent RC relaxation behavior of the device, which could also contribute to the prolonged transient voltage decay.

The prolonged voltage pulse observed in the h-BN/GaN device provides complementary evidence that h-BN integration improves piezopotential preservation and transient signal retention. Together with the enhanced output voltage, current density, and XPS-derived interfacial electronic structure modulation, these results suggest that the h-BN/GaN heterostructure provides a more favorable interfacial environment for maintaining piezoelectric charge generation and signal stability compared with the single GaN device.

**Figure 6:**
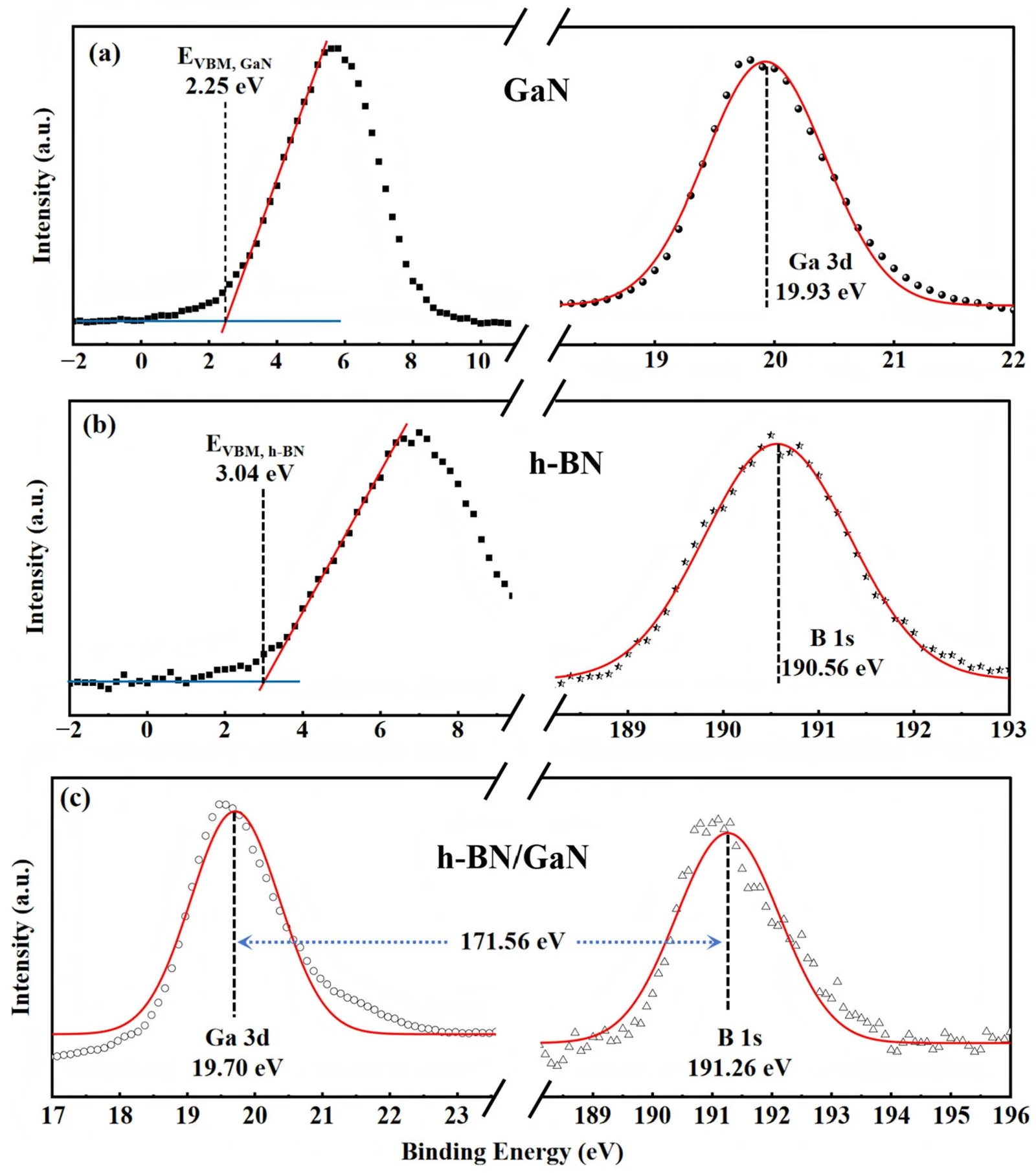
XPS determination of the valence-band and core-level energies of GaN, h-BN, and the h-BN/GaN heterostructure. (**a**) Valence-band spectrum and Ga 3d core-level spectrum of GaN, showing a valence-band maximum (VBM) of 2.25 eV and a Ga 3d binding energy of 19.93 eV. (**b**) Valence-band spectrum and B 1s core-level spectrum of h-BN, with the VBM and B 1s binding energy determined to be 3.04 and 190.56 eV, respectively. The VBM positions were obtained by linear extrapolation of the leading edge of the valence-band spectra to the background baseline. (**c**) High-resolution Ga 3d and B 1s spectra of the h-BN/GaN heterostructure, showing binding energies of 19.70 and 191.26 eV, respectively, corresponding to an interfacial core-level separation of 171.56 eV. These experimentally determined energies were used for subsequent evaluation of the h-BN/GaN band offsets using the Kraut method.

To directly investigate the electronic structure of the h-BN/GaN interface, XPS valence-band and core-level measurements were further performed. As shown in Figure 6a, the valence-band maximum ($E_{VBM}^{GaN}$) and Ga 3d core level ($E_{Ga3d}^{GaN}$) of the GaN reference are located at 2.25 and 19.93 eV, respectively, corresponding to $(E_{Ga3d}-E_{VBM}{)}_{GaN}=17.68$ eV. For h-BN, the VBM ($E_{VBM}^{hBN}$) and B 1s core level ($E_{B1s}^{hBN}$) are located at 3.04 and 190.56 eV, respectively, giving $(E_{B1s}-E_{VBM}{)}_{hBN}=187.52$ eV (Figure 6b). At the h-BN/GaN interface, the Ga 3d ($E_{Ga3d}^{interface}$) and B 1s ($E_{B1s}^{interface}$) core levels are observed at 19.70 and 191.26 eV, respectively, resulting in an interfacial core-level separation ($(E_{B1s}-E_{Ga3d}{)}_{interface}$) of 171.56 eV (Figure 6c). According to the Kraut method [42],

(6) $$\Delta E_{V}=(E_{Ga3d}-E_{VBM}{)}_{GaN}-(E_{B1s}-E_{VBM}{)}_{hBN}+(E_{B1s}-E_{Ga3d}{)}_{interface}$$

the valence-band offset $\Delta E_{V}$ is therefore calculated to be approximately 1.72 eV. Taking the band gaps of GaN and h-BN as 3.40 and 5.90 eV [43,44], respectively, the corresponding conduction-band offset

(7) $$\Delta E_{C}=E_{g}^{hBN}-E_{g}^{GaN}-\Delta E_{V}$$

$\Delta E_{C}$ is approximately 0.78 eV. The resulting band alignment therefore reveals a substantial electron-blocking conduction-band discontinuity at the h-BN/GaN heterointerface.

Notably, the Ga 3d core level shifts from 19.93 eV for the GaN reference to 19.70 eV after formation of the h-BN/GaN interface. The approximately 0.23 eV shift toward lower binding energy is consistent with upward band bending on the n-GaN side of the interface, suggesting depletion-like redistribution of mobile electrons near the heterointerface. The experimentally determined band offset and interfacial band bending thus provide an electronic-structure basis for reducing the availability of free electrons that can compensate strain-induced piezoelectric polarization charges. Accordingly, the enhanced piezoelectric response is attributed to an interfacial-barrier-assisted preservation of the GaN piezopotential. It should be noted that static XPS probes the equilibrium electronic structure rather than the dynamic carrier-screening process itself; therefore, suppression of free-carrier screening is supported by the combined XPS, electrical, transient-response, and piezoelectric measurements.

**Figure 7:**
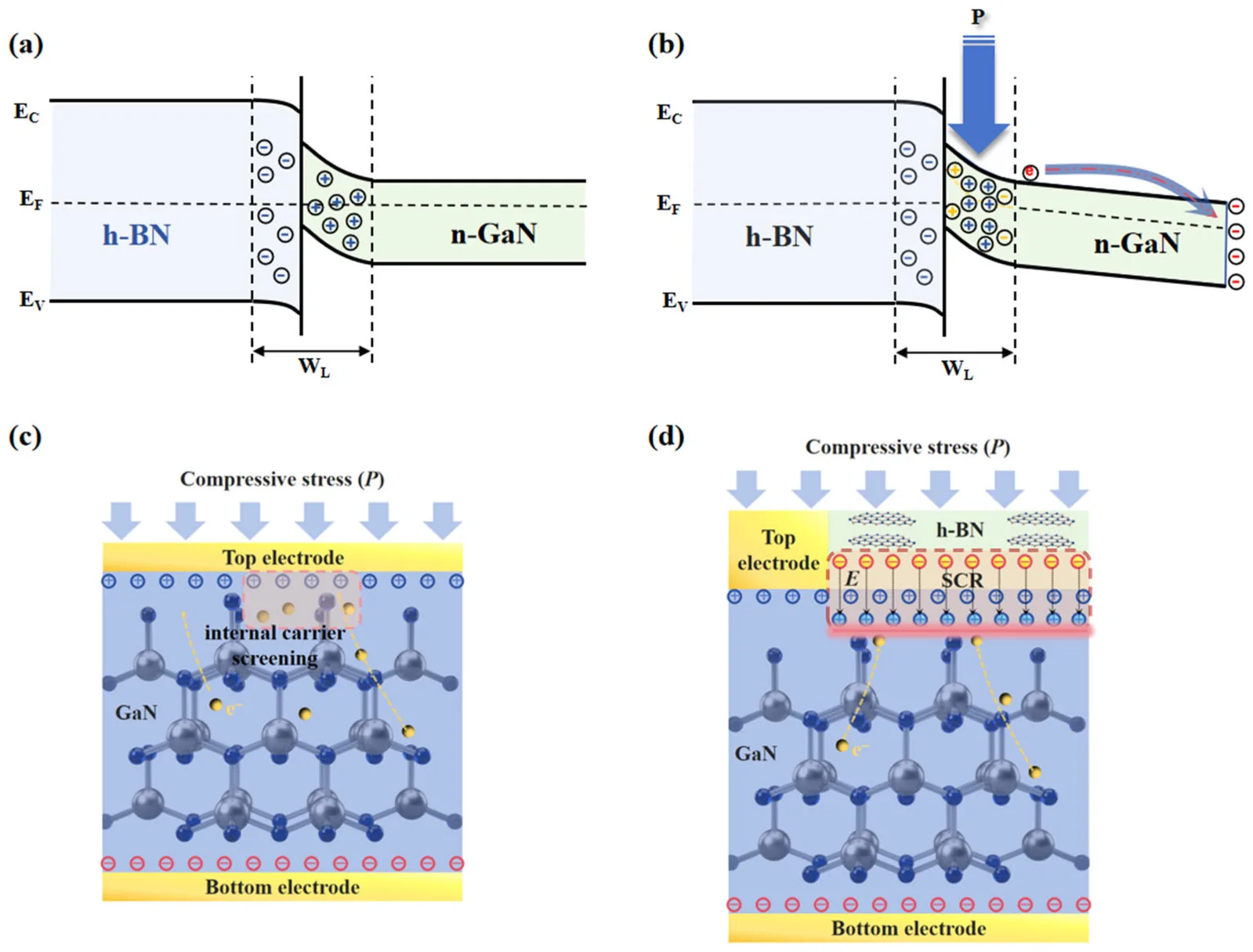
Mechanism of piezopotential preservation and free-carrier screening suppression in the h-BN/GaN heterostructure. (**a**) Equilibrium energy-band diagram of the h-BN/n-GaN heterointerface, showing band bending and the formation of a depletion-like space charge region near the interface. (**b**) Energy-band modulation under compressive stress, where strain-induced piezoelectric polarization charges perturb the interfacial band bending and promote charge redistribution in GaN. (**c**) Schematic illustration of the carrier-screening process in the GaN reference, where mobile electrons rapidly compensate the piezoelectric polarization charges and weaken the effective piezopotential. (**d**) Schematic illustration of the h-BN/GaN device, in which the interfacial space charge region and insulating h-BN layer suppress rapid electron screening and help preserve the strain-induced piezopotential.

Based on the structural, electrical, and piezoelectric characterizations discussed above, the enhancement mechanism of the h-BN/GaN piezoelectric device is schematically illustrated in Figure 7. The key role of h-BN is not only to serve as a thermally stable and electrically insulating surface layer, but also to construct a barrier-type heterointerface with n-GaN, thereby regulating the redistribution of free carriers under mechanical deformation.

Figure 7a shows the equilibrium energy-band diagram of the h-BN/n-GaN heterostructure before external pressure is applied. Since the unintentionally doped GaN layer exhibits weak n-type conductivity, with a measured electron concentration of approximately 5 × 10^16^ cm^−3^, mobile electrons are present in the GaN layer and can participate in charge compensation. After contact with h-BN, interfacial charge redistribution occurs until the Fermi levels tend to align, leading to band bending on the GaN side and the formation of a depletion-like space charge region near the h-BN/GaN interface. In this region, the concentration of mobile electrons is reduced, and an internal electric field is established. This interfacial barrier is consistent with the asymmetric J–V behavior observed in Figure 4a, indicating that carrier transport across the h-BN/GaN interface is no longer purely ohmic but is modulated by the interfacial potential barrier.

When compressive stress is applied, the non-centrosymmetric wurtzite GaN layer generates piezoelectric polarization charges, as illustrated in Figure 7b. In bare n-GaN, these piezoelectric charges can be rapidly compensated by free electrons, resulting in strong carrier screening and a reduced effective piezopotential. In contrast, in the h-BN/GaN heterostructure, the pre-existing depletion-like region near the interface acts as a carrier-depleted protection layer, which suppresses the rapid migration of free electrons toward the piezoelectric polarization charges. As a result, a larger fraction of the strain-induced piezopotential can be preserved. Meanwhile, the generated piezopotential transiently perturbs the original band equilibrium and modulates the interfacial band bending. This strain-induced modulation can drive electron redistribution away from the interfacial region and toward the opposite side of the GaN layer, further strengthening the effective potential difference between the two electrodes.

The difference between the bare GaN and h-BN/GaN devices can be further understood from the carrier-screening models shown in Figure 7c,d. For the GaN reference (Figure 7c), compressive stress induces polarization charges at the GaN surfaces. However, because the GaN layer contains residual free electrons, these electrons can rapidly migrate and neutralize the positive piezoelectric charges near the stressed surface. This internal carrier screening weakens the piezoelectric potential, leading to the relatively low voltage and current-density outputs observed for the reference GaN device in Figure 4b,c. It also explains the shorter transient voltage duration shown in Figure 5a, where the generated voltage decays rapidly after mechanical stimulation.

After h-BN integration, the h-BN/GaN interface forms a space charge region that separates the mobile carriers in GaN from the strain-induced polarization charges, as shown in Figure 7d. The insulating h-BN layer and the interfacial barrier jointly hinder fast electron compensation, allowing the piezoelectric potential to be maintained for a longer time. This mechanism is supported by the enhanced voltage and current-density outputs of the h-BN/GaN device in Figure 4b,c, the larger simulated piezopotential of the h-BN/GaN structure in Figure 4d,e, and the prolonged transient voltage duration in Figure 5b. Therefore, the improved piezoelectric response of the h-BN/GaN device originates from the combined effects of interfacial depletion, suppressed free-carrier screening, and strain-induced barrier modulation.

Overall, the h-BN/GaN heterostructure provides an effective piezotronic interface for preserving and amplifying the piezoelectric potential generated in GaN. The interfacial space charge region reduces rapid electron screening, while the pressure-induced piezopotential dynamically modulates the band bending and charge distribution across the heterointerface. This mechanism accounts for the higher output voltage, larger current-density response, improved voltage retention, and good pressure-dependent behavior of the flexible h-BN/GaN/Cu piezoelectric sensor.

In addition to electromechanical conversion, thermal management is another critical requirement for piezoelectric sensors operating in harsh environments. Since the device consists of micron-scale thin films, conventional bulk thermal-conductivity measurement methods are difficult to apply directly. Raman optothermal analysis was therefore employed to evaluate the local heat-dissipation behavior of the GaN and h-BN/GaN devices by monitoring the temperature- and laser-power-dependent shift in the GaN E_2_(high) phonon mode.

**Figure 8:**
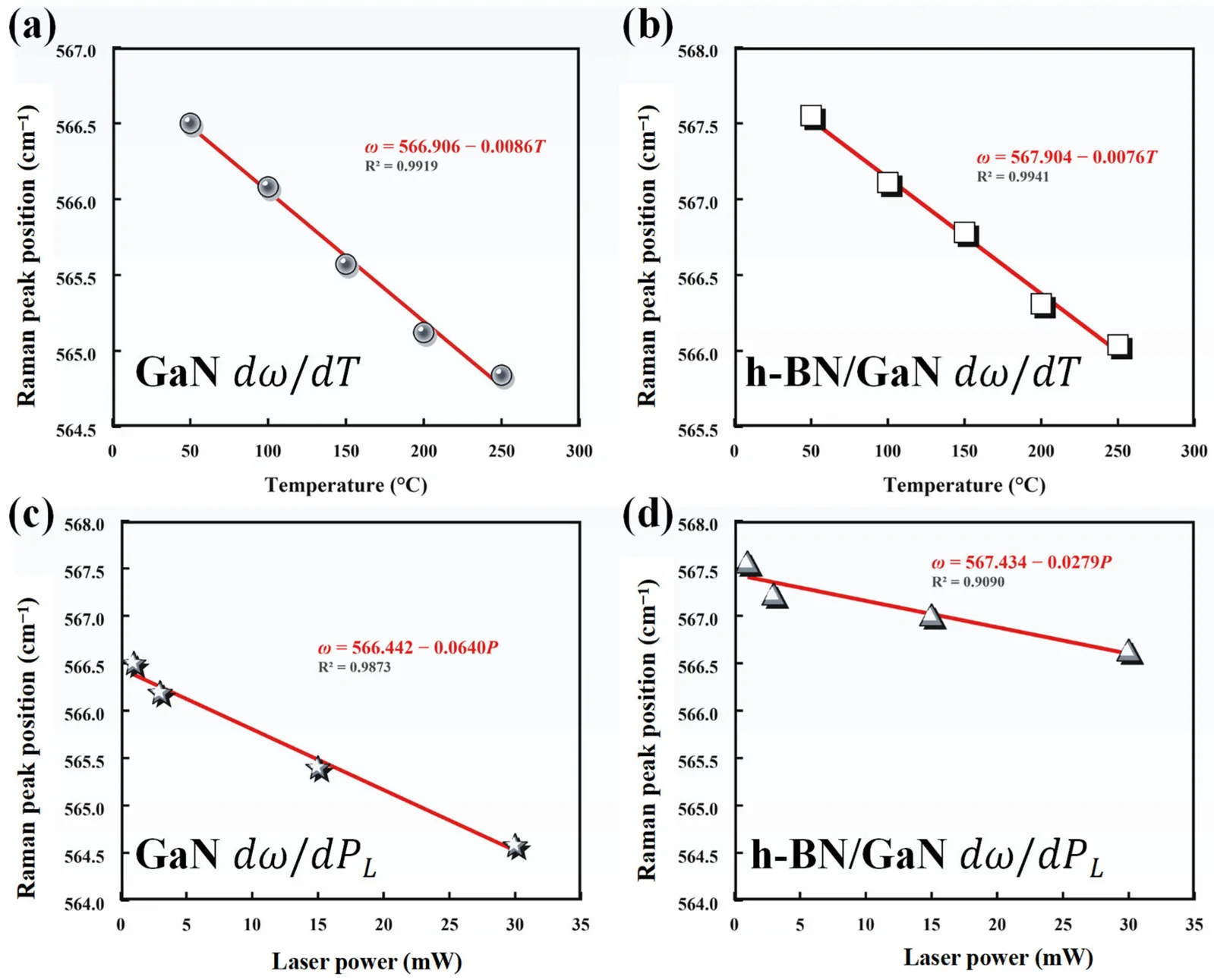
Raman optothermal analysis of the GaN and h-BN/GaN devices. (**a**,**b**) Temperature-dependent shift in the GaN E_2_(high) Raman peak for the bare GaN and h-BN/GaN devices, respectively. The fitted temperature coefficients are (−0.0086) and (−0.0076) cm^−1^/°C. (**c**,**d**) Laser-power-dependent shift in the GaN E_2_(high) Raman peak for the bare GaN and h-BN/GaN devices, respectively. The reduced power coefficient of the h-BN/GaN device indicates suppressed laser-induced local heating and improved effective heat dissipation after h-BN integration.

Figure 8a,b show the temperature-dependent Raman peak positions of the GaN E2(high) mode for the bare GaN and h-BN/GaN devices, respectively. In both cases, the Raman peak exhibits a nearly linear red shift with increasing temperature, which is mainly attributed to lattice thermal expansion and anharmonic phonon–phonon interactions. The fitted temperature coefficients are (−0.0086) cm^−1^/°C for GaN and (−0.0076) cm^−1^/°C for h-BN/GaN, indicating comparable thermally induced phonon softening in the two structures. These temperature coefficients provide the necessary calibration for converting laser-induced Raman shifts into local temperature variations.

The laser-power-dependent Raman measurements are shown in Figure 8c,d. As the laser power increases, local optical heating causes a red shift in the GaN E_2_(high) mode. For the GaN reference, the fitted power coefficient is (−0.0640) cm^−1^/mW, whereas the h-BN/GaN device shows a much smaller value of (−0.0279) cm^−1^/mW. The significantly reduced magnitude of *dω*/*dP_L_* indicates that, under the same laser excitation, the GaN layer in the h-BN/GaN device experiences a smaller local temperature rise. This result suggests that the introduction of h-BN facilitates more efficient heat spreading and suppresses localized self-heating in the active piezoelectric region.

According to Raman optothermal analysis, the effective thermal transport capability can be qualitatively compared using the relationship (K ∝ (*dω*/*dT*)/*(dω*/*dP_L_*)) [45]. The relative thermal response extracted from the Raman optothermal measurements indicates that the h-BN/GaN heterostructure exhibits a lower local temperature rise compared with the GaN reference under identical optical excitation conditions. This improvement is attributed to the high thermal stability and heat-spreading capability of h-BN, together with its intimate interfacial contact with GaN, as confirmed by the structural characterization in Figure 2. The improved thermal dissipation can reduce heat accumulation in the GaN piezoelectric layer, thereby helping to maintain stable polarization and output signals under elevated-temperature operation.

These Raman optothermal results provide additional evidence that h-BN integration not only enhances the piezoelectric output through interfacial charge regulation, as discussed in Figure 4, Figure 5, Figure 6 and Figure 7, but also improves the thermal-management characteristics of the device. Such combined electromechanical and thermal advantages are essential for reliable operation of the h-BN/GaN piezoelectric sensor in harsh environments.

**Figure 9:**
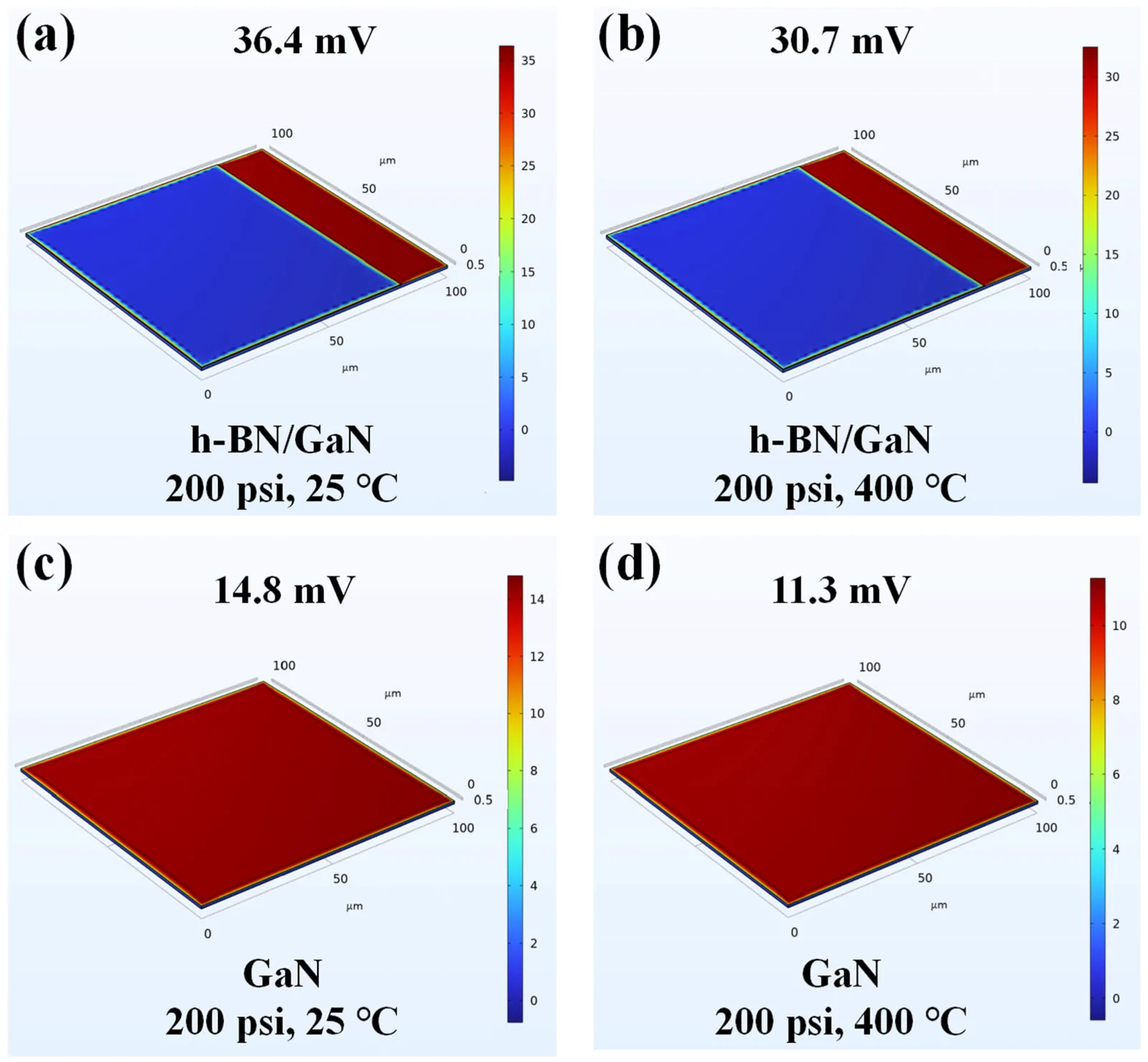
Temperature-dependent COMSOL simulation of piezopotential distributions under an input pressure of 200 psi. (**a**,**b**) Simulated piezopotential distributions of the h-BN/GaN structure at 25 °C and 400 °C, showing maximum output voltages of 36.4 and 30.7 mV, respectively. (**c**,**d**) Simulated piezopotential distributions of the GaN reference at 25 °C and 400 °C, showing maximum output voltages of 14.8 and 11.3 mV, respectively. The h-BN/GaN structure exhibits a higher output voltage and lower thermal attenuation than the GaN reference, indicating improved piezopotential preservation under elevated-temperature conditions.

To evaluate the temperature dependence of the electrostatic response, finite-element calculations were performed under an identical nominal pressure of 200 psi while the temperature-dependent dielectric and electromechanical parameters were varied from 298.15 to 673.15 K. At room temperature, the h-BN/GaN structure exhibits a maximum modeled potential of approximately 36.4 mV, compared with 14.8 mV for the GaN reference (shown in Figure 9).

The higher simulated potential indicates that the introduction of h-BN modifies the electrostatic response of the heterostructure and promotes more effective potential retention under mechanical excitation. This behavior is consistent with the experimentally observed enhancement of the h-BN/GaN piezoelectric output.

At 400 °C, the modeled potentials decrease to approximately 30.7 mV for h-BN/GaN and 11.3 mV for GaN. The corresponding voltage attenuation from room temperature to 400 °C is approximately 15.7% and 23.6%, respectively. These results indicate that the h-BN/GaN structure exhibits improved thermal retention of the electrostatic/piezopotential response compared with the GaN reference.

The reduced temperature-induced degradation suggests that the h-BN interfacial layer contributes to maintaining the electromechanical response of the heterostructure under harsh thermal conditions. This trend agrees well with the experimental observation that the h-BN/GaN device preserves higher output performance at elevated temperatures.

Therefore, the simulation results provide further support for the enhancement mechanism proposed in Figure 7. The h-BN/GaN heterostructure not only increases the piezopotential output under the same pressure loading, but also mitigates thermal degradation of the voltage response. These advantages are critical for piezoelectric sensing applications in harsh environments where both mechanical pressure and elevated temperature are present.

**Figure 10:**
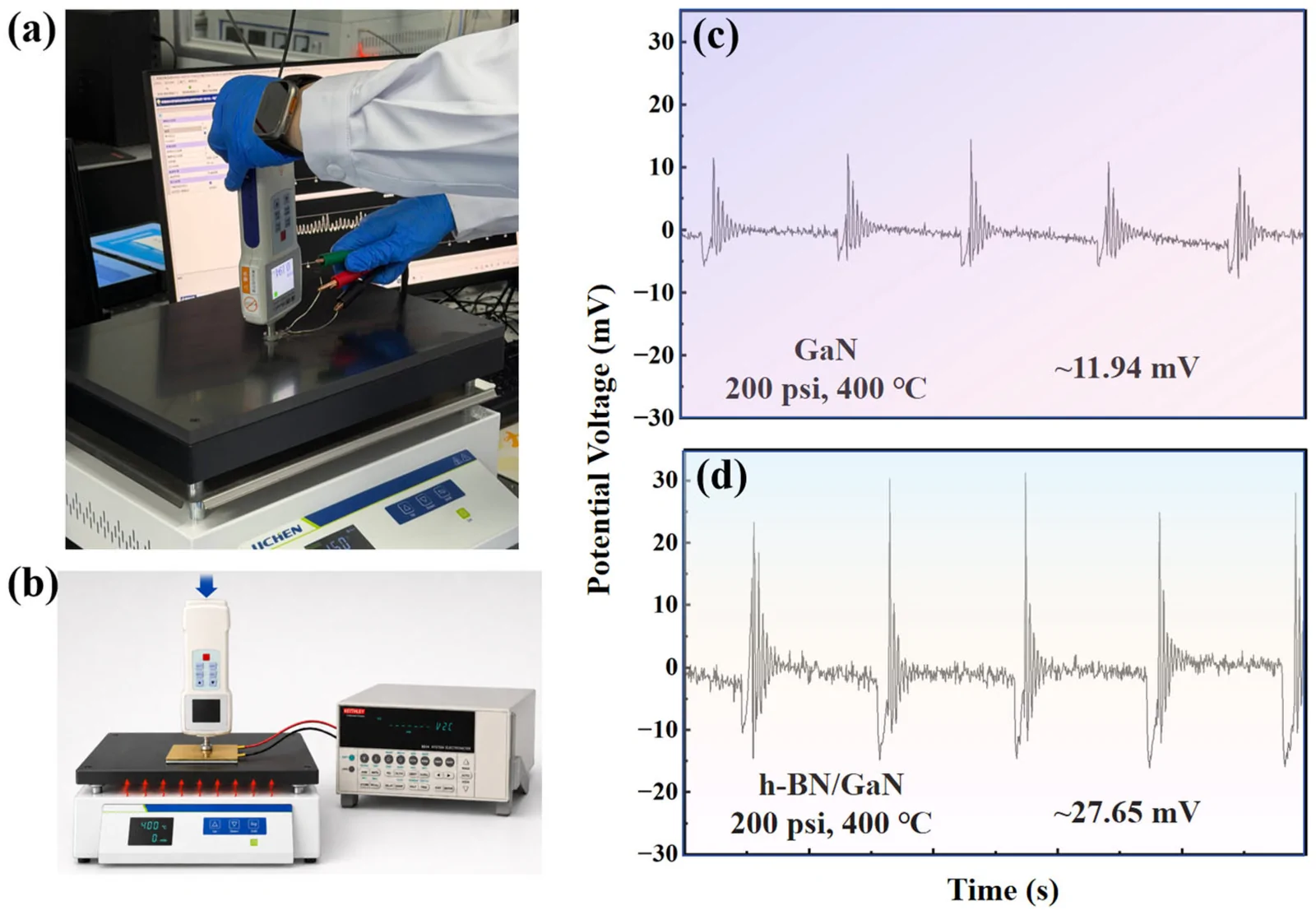
High-temperature piezoelectric response of the GaN and h-BN/GaN devices under coupled thermal–mechanical conditions. (**a**) Photograph of the experimental setup for pressure-response measurement on a heating stage. (**b**) Schematic illustration of the high-temperature pressure-testing configuration, including the heating stage, force gauge, device, and electrometer. (**c**) Output voltage response of the GaN reference under 200 psi at 400 °C, showing an amplitude of approximately 11.94 mV. (**d**) Output voltage response of the h-BN/GaN device under 200 psi at 400 °C, showing an enhanced amplitude of approximately 27.65 mV. The higher output voltage of the h-BN/GaN device confirms its improved piezoelectric performance under high-temperature conditions.

To further verify the practical applicability of the h-BN/GaN piezoelectric device under harsh environments, high-temperature pressure-response measurements were performed at 400 °C, as shown in Figure 10. Figure 10a presents the photograph of the experimental setup, in which a force gauge was used to apply periodic pressure to the flexible device placed on a heating stage. A schematic illustration of the measurement configuration is shown in Figure 10b. During testing, the device was subjected to repeated compressive loading while the output voltage was recorded by an electrometer. This configuration enables direct evaluation of the piezoelectric response under coupled high-temperature and mechanical-pressure conditions.

Figure 10c,d compare the output voltage signals of the bare GaN and h-BN/GaN devices under 200 psi at 400 ℃. The GaN reference exhibits a relatively weak voltage response, with an output amplitude of approximately 11.94 mV. In contrast, the h-BN/GaN device maintains a much higher output voltage of approximately 27.65 mV under the same testing condition. The output voltage of the h-BN/GaN device is therefore about 2.32 times that of the GaN reference at 400 °C, demonstrating that the h-BN/GaN heterostructure remains effective in enhancing piezoelectric output even under elevated-temperature operation.

The experimental high-temperature results are also consistent with the temperature-dependent finite-element simulation shown in Figure 9, where the simulated output voltages at 400 ℃ were 11.3 mV for bare GaN and 30.7 mV for h-BN/GaN. Although slight differences between simulation and experiment are expected because of nonideal contact, actual pressure distribution, thermal gradients, electrode resistance, and packaging effects, the overall trend and voltage magnitude agree well. This agreement further supports the proposed mechanism that h-BN integration can help preserve the piezopotential in GaN by suppressing free-carrier screening and stabilizing the interfacial electric field.

More importantly, the h-BN/GaN device exhibits repeatable voltage pulses at 400 ℃, indicating that the all-inorganic heterostructure maintains stable electromechanical conversion under harsh thermal–mechanical conditions. The stable voltage pulses obtained under repeated loading demonstrate good cycle-to-cycle repeatability and reversible piezoelectric response of the representative h-BN/GaN device. It should be emphasized that these repeated measurements characterize the repeatability of an individual device and are not intended to establish device-to-device statistical reproducibility. Combined with the Raman optothermal analysis in Figure 8, which suggests improved heat-dissipation capability after h-BN integration, these high-temperature pressure-response results confirm that the h-BN/GaN/Cu device possesses strong potential for pressure sensing in high-temperature industrial environments.

**Figure 11:**
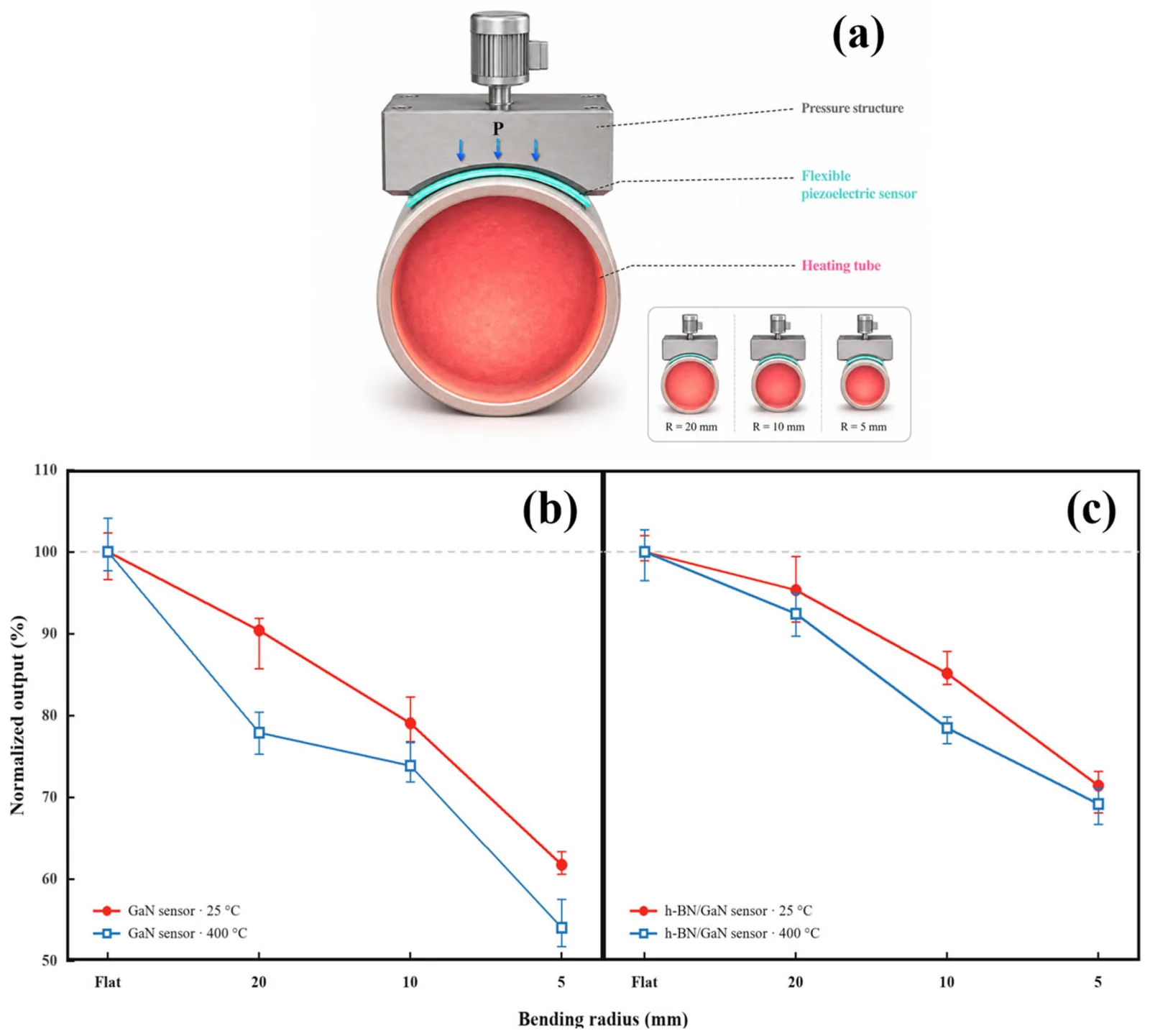
Mechanical flexibility and high-temperature operational stability of GaN and h-BN/GaN piezoelectric sensors. (**a**) Schematic illustration of the curvature-controlled bending and heating platform. Different bending radii were achieved using interchangeable heating tubes, while the pressure structure provided conformal loading. (**b**,**c**) Normalized output retention of GaN and h-BN/GaN devices under different bending radii at room temperature and 400 °C. Each data point represents the average value obtained from five independently fabricated devices after 1000 bending cycles and 1 h continuous piezoelectric operation. Error bars indicate the maximum and minimum values among the tested devices.

To further evaluate the mechanical flexibility and thermal operational stability of the flexible piezoelectric sensors, a curvature-controlled high-temperature bending platform was developed, as illustrated in Figure 11a. Different bending radii were achieved by replacing cylindrical heating tubes with different diameters, while a matching pressure head fabricated by mechanical machining was used to ensure conformal contact between the loading structure and the flexible sensor. The flexible h-BN/GaN and GaN reference devices were subjected to repeated bending deformation and continuous piezoelectric operation under controlled temperature conditions.

For each bending radius and temperature condition, five independently fabricated devices were tested. The normalized output was calculated based on the average peak voltage after 1000 bending cycles and 1 h continuous pressure loading. The error bars represent the maximum and minimum values among the five tested devices, reflecting the device-to-device variation.

As shown in Figure 11b, the GaN reference devices exhibit a gradual decrease in normalized output with decreasing bending radius. At room temperature, the normalized output decreases from 100% under flat conditions to approximately 90%, 79%, and 62% at bending radii of 20, 10, and 5 mm, respectively. After high-temperature operation at 400 °C, the output retention further decreases, reaching approximately 78%, 74%, and 54% at the corresponding bending radii. In comparison, the h-BN/GaN heterostructure demonstrates improved mechanical and thermal stability. At room temperature, the output retention remains approximately 95%, 85%, and 71% at bending radii of 20, 10, and 5 mm, respectively. More importantly, after continuous operation at 400 °C, the h-BN/GaN device maintains higher output retention of approximately 92%, 78%, and 69% under the same bending conditions.

The improved retention under bending and elevated temperature is attributed to the enhanced interfacial stability and mechanical compliance provided by the h-BN/GaN heterostructure. The atomically thin h-BN interfacial layer can accommodate local strain redistribution while maintaining stable electronic coupling at the heterointerface, which contributes to improved robustness during combined mechanical and thermal stresses. These results demonstrate that the h-BN/GaN flexible piezoelectric sensor maintains stable electromechanical performance under repeated bending deformation and elevated-temperature operation, providing further evidence of its suitability for harsh-environment sensing applications.

**Figure 12:**
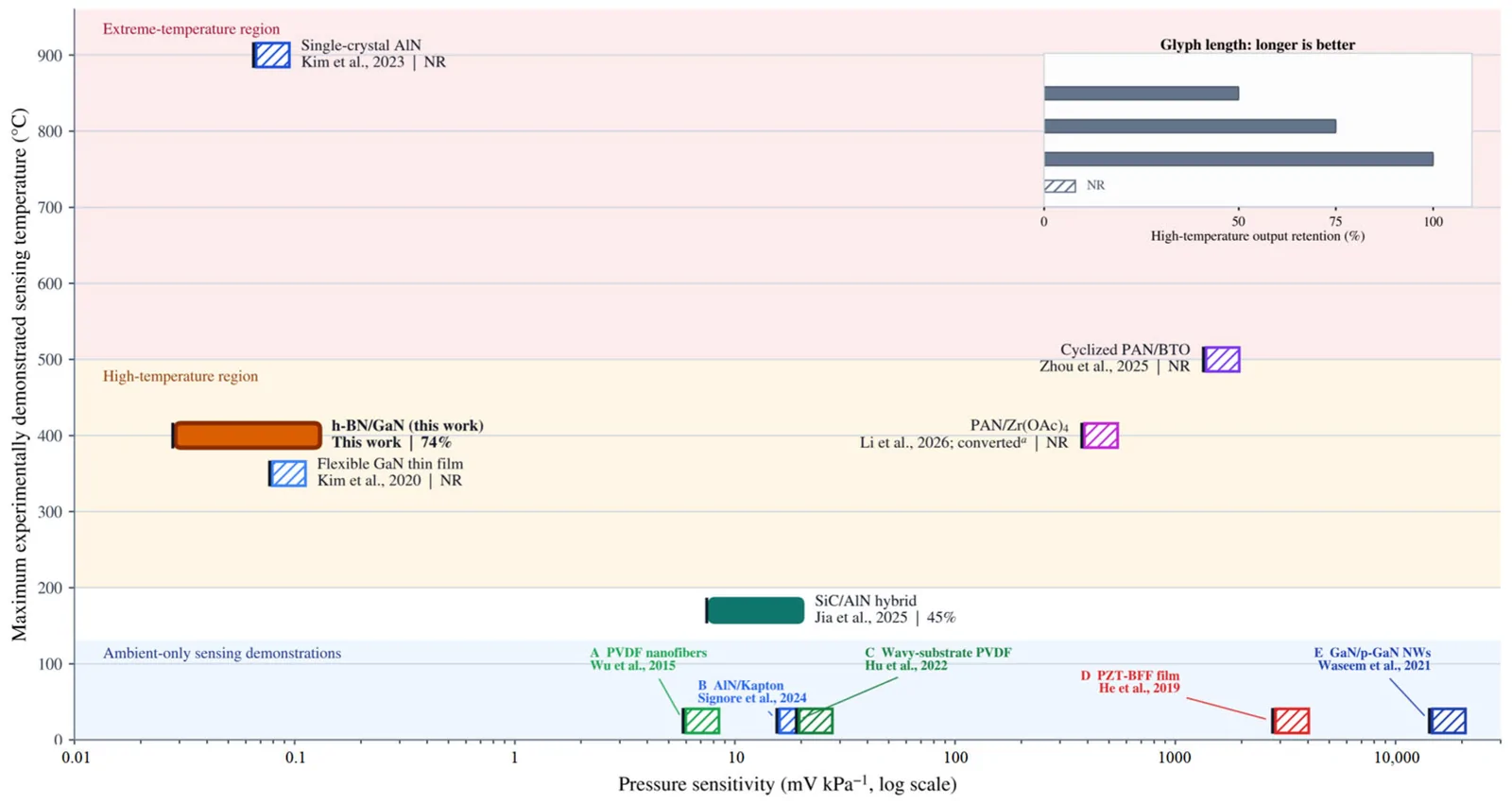
Benchmarking of representative piezoelectric sensors for flexible and harsh-environment applications [1,2,13,46,47,48,49,50,51,52]. The comparison summarizes experimentally demonstrated operating temperatures and pressure sensitivities of reported polymer-, ceramic-, and wide-bandgap semiconductor-based piezoelectric devices. The inset compares high-temperature output retention of representative devices, highlighting the combined high-temperature capability and operational stability of the h-BN/GaN sensor developed in this work.

To further clarify the advantages of the proposed h-BN/GaN heterostructure, a quantitative comparison with previously reported piezoelectric sensors was performed, as summarized in Figure 12. The comparison includes representative flexible and harsh-environment piezoelectric devices based on polymer, ceramic, and wide-bandgap semiconductor materials, considering key parameters including experimentally demonstrated operating temperature, pressure sensitivity, output retention, device architecture, and mechanical flexibility.

Conventional flexible piezoelectric materials such as PVDF-based devices generally exhibit excellent mechanical compliance and high pressure sensitivity; however, their practical application in high-temperature environments is limited by the thermal instability of polymer matrices. Conversely, ceramic piezoelectric materials and wide-bandgap semiconductor piezoelectric sensors, including PZT-, AlN-, SiC-, and GaN-based devices, provide improved thermal tolerance but are commonly fabricated on rigid substrates or bulk structures, limiting their mechanical adaptability.

Recent advances in flexible GaN-based piezoelectric sensors have demonstrated the feasibility of combining semiconductor piezoelectricity with flexible substrates. However, maintaining stable piezoelectric output under elevated temperatures remains challenging because of carrier compensation, interfacial charge loss, and thermomechanical instability. As shown in Figure 12, the present h-BN/GaN heterostructure simultaneously achieves high-temperature operation at 400 °C and flexible thin-film integration, while maintaining approximately 74% output retention after high-temperature operation. This combination distinguishes the present device from previously reported flexible piezoelectric sensors, which typically exhibit either high sensitivity at ambient conditions or high-temperature capability without mechanical flexibility.

The enhanced performance originates from the synergistic role of the h-BN/GaN heterointerface. Unlike conventional GaN devices that mainly rely on the intrinsic piezoelectric response of GaN, the introduction of h-BN provides an electrically and mechanically stable interfacial layer, which modifies the local charge environment, improves piezopotential preservation, and enhances structural robustness under combined thermal and mechanical stresses. Therefore, the key advancement of this work is not only the extension of the operating temperature of flexible GaN-based piezoelectric sensors, but also the realization of an integrated strategy combining high-temperature stability, mechanical flexibility, and reliable piezoelectric output.

## 4. Conclusions

In summary, a flexible all-inorganic h-BN/GaN/Cu piezoelectric pressure sensor was designed and fabricated for harsh-environment sensing applications. The h-BN thin film was directly grown on GaN/Si by PECVD, followed by backside Si removal, electrode deposition, and transfer onto a flexible Cu foil substrate. Cross-sectional HRTEM, XRD, Raman, and XPS characterizations confirmed the successful formation of a compact h-BN/GaN heterostructure with interfacial electronic structure modulation. Raman analysis of the GaN E2(high) mode further indicated that release from the rigid Si substrate and flexible integration reduced residual tensile stress and improved local strain uniformity in the GaN layer.

Electrical and piezoelectric measurements demonstrated that h-BN integration significantly enhanced the output performance of the GaN-based sensor. Compared with the GaN reference device, the h-BN/GaN device exhibited modified interfacial transport behavior, higher voltage output, enhanced transient current response, and improved voltage-retention characteristics. These improvements are proposed to originate from the electronic and mechanical modulation introduced by the h-BN/GaN interface, which may contribute to regulating carrier compensation processes and preserving strain-induced piezoelectric polarization charges. Finite-element simulations further revealed modified electrostatic potential distribution after h-BN incorporation, consistent with the experimentally observed enhancement in piezoelectric performance.

In addition to electromechanical enhancement, Raman optothermal analysis indicated improved relative/local thermal response after h-BN integration. Temperature-dependent simulations, high-temperature pressure-response measurements, and combined bending–thermal stability tests further demonstrated the operational robustness of the heterostructure device. At 400 °C and 200 psi, the h-BN/GaN sensor maintained an output voltage of approximately 27.65 mV, which was about 2.32 times higher than that of the GaN reference under identical conditions. These results highlight the potential of h-BN/GaN heterostructure engineering as an effective strategy for developing flexible piezoelectric sensors capable of operating under coupled high-temperature and mechanical environments.

## Figures and Tables

**Table 1:** Main parameters and boundary conditions used in the COMSOL simulation.

Category	Parameter	GaN	h-BN
Geometry	Lateral dimension	100 × 100 µm^2^	100 × 100 µm^2^
Geometry	Thickness	1.0 µm	0.2 µm
Dielectric	ε_x_, ε_γ_	9.5	6.93
Dielectric	ε_z_	10.4	3.76
Piezoelectric	e_33_	0.73 C m^−2^	—
Elastic	C_33_	398 GPa	—
Temperature correction	α_ε_	3.0 × 10^−4^ K^−1^	1.0 × 10^−4^ K^−1^
Temperature correction	α_C33_	7.0 × 10^−5^ K^−1^	—
Temperature correction	α_e33_	1.5 × 10^−4^ K^−1^	—
Loading	Nominal pressure	50–200 psi	Identical for both structures
Temperature	Simulation range	298.15–673.15 K	Identical for both structures
Electrical BC	Bottom electrode	Ground	—
Electrical BC	External bias	0 V	—
Other surfaces	Electrical condition	Electrical insulation	Electrical insulation

## Data Availability

The original contributions presented in this study are included in the article. Further inquiries can be directed to the corresponding author.
